# Co-circulation and genetic characterization of genotype I and II feline bocavirus strains in domestic cats from Northern Vietnam

**DOI:** 10.14202/vetworld.2025.1590-1598

**Published:** 2025-06-16

**Authors:** Hieu Van Dong, Giang Thi Huong Tran, Yen Hoang Thi Nguyen, Thiet Chi Ngo, Amonpun Rattanasrisomporn, Chaiwat Boonkaewwan, Dao Anh Tran Bui, Jatuporn Rattanasrisomporn

**Affiliations:** 1 Department of Veterinary Public Health, Faculty of Veterinary Medicine, Vietnam National University of Agriculture, Trau Quy Town, Gia Lam District, Hanoi, Vietnam; 2 Department of Veterinary Microbiology and Infectious Diseases, Faculty of Veterinary Medicine, Vietnam National University of Agriculture, Trau Quy Town, Gia Lam District, Hanoi, Vietnam; 3 Department of Veterinary Parasitology, Faculty of Veterinary Medicine, Vietnam National University of Agriculture, Trau Quy Town, Gia Lam District, Hanoi, Vietnam; 4 Interdisciplinary of Genetic Engineering and Bioinformatics, Graduate School, Kasetsart University, Bangkok, Thailand; 5 Department of Companion Animal Clinical Sciences, Faculty of Veterinary Medicine, Kasetsart University, Bangkok, Thailand; 6 Department of Veterinary Pathology, Faculty of Veterinary Medicine, Vietnam National University of Agriculture, Trau Quy Town, Gia Lam District, Hanoi, Vietnam

**Keywords:** domestic cats, feline bocavirus, genotype, molecular epidemiology, NS1 gene, Vietnam

## Abstract

**Background and Aim:**

Feline bocavirus (FBoV), a member of the *Parvoviridae* family, has been implicated in gastrointestinal and respiratory conditions in domestic cats. Despite increasing global recognition, the molecular epidemiology of FBoV in Vietnamese animal populations remains largely unexplored. This study aimed to detect and genetically characterize FBoV strains circulating among domestic cats in Northern Vietnam to better understand their genotypic diversity and potential clinical relevance.

**Materials and Methods:**

A total of 166 fecal samples were collected from domestic cats of varying age, sex, and clinical status across four provinces in Northern Vietnam between 2022 and 2023. DNA was extracted and screened for FBoV using conventional polymerase chain reaction targeting the non-structural (NS)-1 gene. Positive samples were subjected to Sanger sequencing, and partial NS1 sequences were analyzed using MEGA X for phylogenetic inference. Recombination analysis was performed using RDP 4.0, and statistical significance was assessed using Fisher’s exact test.

**Results:**

FBoV DNA was detected in 4 of 166 samples (2.41%), including one from a diarrheic cat and three from healthy cats. Phylogenetic analysis of the partial NS1 gene revealed that three strains belonged to genotype I and one to genotype II, all showing close genetic similarity to Chinese strains. Nucleotide identities among Vietnamese strains ranged from 64.68% to 99.57%. No recombination events were observed among the detected strains. FBoV was detected across age groups and both sexes, without significant associations. Co-infections with other enteric viruses (feline coronavirus, feline panleukopenia virus, feline astrovirus, and feline kobuvirus) were not observed in the FBoV-positive samples.

**Conclusion:**

This study provides the first molecular evidence of co-circulating FBoV genotypes I and II in domestic cats in Vietnam, indicating viral genetic diversity and suggesting possible regional transmission routes linked to neighboring countries. While FBoV was present in both symptomatic and asymptomatic cats, its clinical significance remains inconclusive. The findings underscore the need for expanded surveillance, complete genome analyses, and investigation into FBoV’s pathogenic potential and co-infection dynamics in the feline population. These data will be instrumental in shaping future diagnostic and control strategies for feline viral enteritis in Vietnam.

## INTRODUCTION

Bocavirus (BoV), a member of the *Parvoviridae* family, has been detected in a broad range of host species, including dogs, cats, humans, sea lions, pigs, and cattle [1–5]. In animals, BoV infection has been associated with gastrointestinal and respiratory disorders, such as diarrhea in dogs and hemorrhagic enteritis in cats, while in humans and piglets, it is often linked to respiratory and enteric illnesses [2, 6–8]. Feline bocavirus (FBoV) was first identified in 2011 in Hong Kong, China, from kidney, blood, and fecal samples of asymptomatic stray cats [9]. Since then, FBoV has been reported in several countries, including the United States, Japan, China, Portugal, and Thailand, and has been found in both clinically healthy and diseased cats [6, 10–13]. To date, three genotypes of FBoV (FBoV-1, FBoV-2, and FBoV-3) have been identified globally in feline populations [14].

FBoV belongs to the genus BoV within the family *Parvoviridae* [15]. It is a non-enveloped virus, approximately 25–26 nm in diameter, with an isometric icosahedral morphology and a linear single-stranded DNA genome of ~5.0 kilobase pairs [16]. The genome contains three open reading frames (ORFs): ORF1 encodes the non-structural protein NS1, ORF2 encodes the capsid proteins VP1 and VP2, and ORF3 encodes the nuclear phosphoprotein NP1 [16]. The VP1 protein plays a pivotal role in viral antigenicity, pathogenicity, and host cell invasion, and mutations in this gene may alter its structural conformation, influencing viral virulence [4, 17]. VP2 is implicated in determining host range and exhibits notable genetic variability among FBoV-1 strains [18, 19]. The NP1 protein is unique to bocaviruses and is thought to facilitate viral replication and enhance capsid protein expression [18, 20]. Variations in NP1 have been proposed to contribute to the emergence of novel genotypes [21]. Similarly, NS1 is highly conserved and functionally critical in viral replication, making it a valuable target for molecular characterization of FBoV strains [20–22].

Human bocavirus (HBoV) has also been reported in Vietnam, particularly among children aged 12–24 months hospitalized with acute respiratory symptoms. In these cases, HBoV infection was associated with severe outcomes such as hypoxia and pneumonia and extended hospitalization periods. Genetic analyses confirmed the presence of HBoV-1 strains [3, 23].

Despite growing international interest in FBoV and its implications for feline health, the epidemiology and molecular diversity of FBoV remain underexplored in many parts of Southeast Asia, including Vietnam. To date, most studies have focused on FBoV detection and genotyping in cats from China, Japan, the United States, and select European countries, with limited data available from the Vietnamese context. Although HBoV infections have been documented in Vietnamese pediatric populations, suggesting the presence and transmission of BoV in the region, there has been no published data on the occurrence or molecular characterization of FBoV in domestic cats in Vietnam. This paucity of surveillance and genomic data presents a critical barrier to understanding the evolutionary trajectory, pathogenic potential, and regional transmission dynamics of FBoV. In addition, the absence of genotype-specific studies undermines efforts to assess the zoonotic potential of FBoV, mon-itor recombination events, or establish genotype-pathogenicity correlations, especially in relation to co-infections with other feline enteric viruses.

This study aimed to bridge the aforementioned knowledge gap by conducting the first molecular investigation of FBoV among domestic cats in Northern Vietnam. Specifically, we sought to detect the presence of FBoV using polymerase chain reaction (PCR) methods and to genetically characterize the circulating strains through partial NS1 gene sequencing. By analyzing nucleotide identity and phylogenetic clustering, we aimed to determine the genotypes present and assess their genetic relatedness to previously reported strains. Furthermore, we evaluated the infection status of cats with varying clinical presentations (healthy vs. diarrheic) and assessed the potential presence of co-infecting viral pathogens. The outcomes of this study are expected to contribute foundational data for future surveillance, inform diagnostic development, and enhance under-standing of the molecular epide-miology of FBoV in Vietnam and the broader Southeast Asian region.

## MATERIALS AND METHODS

### Ethical approval and Informed consent

All procedures involving animal handling and sample collection were conducted in strict accordance with the ethical guidelines of the Vietnam National University of Agriculture (VNUA). The study protocol was approved by the University’s Committee on Animal Research and Ethics (Approval No. CARE-2023/08). Written informed consent was obtained from all cat owners before sample collection, and the objectives and procedures of the study were clearly explained. Sampling was performed in a manner that minimized stress and discomfort, in compliance with international standards for animal welfare.

### Study period and location

This study was conducted between May 2022 and December 2023 across four northern Vietnamese provinces – Hanoi, Hung Yen, Bac Giang, and Ha Nam.

### Sample collection

A total of 166 fresh fecal samples were collected from domestic cats. The sampled cats ranged in age from 3 to 24 months and included various breeds and clinical conditions (healthy, gastrointestinal symptoms, or other signs). Rectal swabs were obtained using sterile techniques from both clinically healthy and symptomatic animals. All samples were transported on ice to the VNUA laboratory and stored at −80°C until further processing.

### DNA extraction and PCR detection

Genomic DNA was extracted using the Viral Gene-spin™ DNA/RNA Extraction Kit (Intron, Seoul, Korea), following the manufacturer’s protocol. DNA concentration and purity were assessed through spectrophotometry (Nanodrop). Detection of FBoV was performed using conventional PCR with the FBoV-F/R primer pair (Table 1) targeting the viral genome [19, 21, 24–27]. Each PCR run included a known FBoV-positive sample as a positive control and PCR-grade water as a negative control to ensure assay specificity and rule out contamination.

**Table 1:** Primers used in this study.

Name of primer	Nucleotide sequence (5’-3’)	PCR product	Reference
FKoV-3D-F	CTCCGCCCCACCGCTAAGG	530	[19]
FKoV-3D-R	GGGGGTTCCGTTGCGTAGATGA		
FBoV1-NS1F	TTTGGGGCTGAAGTCTGCTATGC	705	[21]
FBoV1-NS1R	ATGCGGCTGAGATGTACCTTGACC		
FBoV2-NS1F	TTCGCGGATCCAGCATACACCTAC	963	[21]
FBoV2-NS1R	CTCACAACGGCGCAAGCAGTCTA		
FBoV-F	AGAACCRCCRATCACARTCCACT	465	[24]
FBoV-R	TGGCRACCGCYAGCATTTCA		
FCoV-P205	GGCAACCCGATGTTTAAAACTGG	223	[25]
FCoV-P211	CACTAGATCCAGACGTTAGCTC		
FeAstV-F	GCGGATTGGGCATGGTTTAGA	645	[26]
FeAstV-R	ACCCCTCGTTTGGATCGTTACCT		
FPV-F	TGCCTCAATCTGAAGGAGCT	250	[27]
FPV-F	TTTCATCTGTTTGCGCTCCC		

FBoV=Feline bocavirus, FCoV=Feline coronavirus, FeAstV=Feline astrovirus, FKoV=Feline kobuvirus, FPV=Feline panleukopenia virus, PCR=Polymerase chain reaction

Additional PCR assays were conducted to screen for other feline enteric viruses, including feline coronavirus (FCoV) [25], feline astrovirus (FeAstV) [26], feline kobuvirus (FKoV) [19], and feline panleukopenia virus (FPV) [27]. The annealing temperature was set to 55°C for FBoV-F/R and 60°C for both FBOV1-NS1F/NS1R and FBOV2-NS1F/NS1R primer sets. Amplified products were separated through 1.5% agarose gel electrophoresis stained with ethidium bromide and visualized under ultraviolet light using a 100 bp DNA ladder (Promega) as a size reference.

### Partial NS1 gene amplification and sequencing

Due to the limited availability of complete FBoV genome sequences in GenBank, amplification of the highly variable partial NS1 gene was carried out on FBoV-positive samples using genotype-specific primers (FBOV1-NS1F/NS1R and FBOV2-NS1F/NS1R) [21]. PCR was conducted with the following thermocycling pro-file: Initial denaturation at 94°C for 5 min, followed by 35 cycles of 94°C for 60 s, 50°C for 60 s, and 72°C for 60 s, with a final extension at 72°C for 7 min. PCR products were purified using the GeneClean® II Kit (MP Biomedicals), and successful recovery was confirmed by agarose gel electrophoresis. Sequencing of the purified amplicons was performed bidirectionally using Sanger sequencing at 1^st^ BASE (Malaysia).

### Bioinformatic and phylogenetic analyses

Sequence alignment was performed using ClustalW in BioEdit version 7.2.5. Homology searches were conducted using BLAST against GenBank entries (accessed March 2024), and genetic distances were calculated using GENETYX (Genetyx Corp., Tokyo, Japan). A total of 53 reference sequences representing three FBoV genotypes were retrieved from GenBank for phylogenetic comparison (Table 2). A phylogenetic tree was constructed using the maximum likelihood method in MEGA X software, employing the Tamura-Nei model with 1,000 bootstrap replicates to assess tree robustness. All newly generated seque-nces were submitted to GenBank (Accession Nos. PP236855–PP236858).

**Table 2:** Description of FBoV and FPV strains used in this study.

GenBank accession number	Strain	Location	Source	Year	Genotype	Virus
KF792837.1	POR1	Portugal	Feline	2012	II	FBoV
LC148406.1	KU-58	Japan	Feline	2015	II	FBoV
LC148407.1	KU-61	Japan	Feline	2015	II	FBoV
MK671205.1	CBV3/16CC0803	China	Feline	2016	I	FBoV
MH155929.1	16CC1103	China	Feline	2016	II	FBoV
MH155933.1	16JZ0613	China	Feline	2016	II	FBoV
MK671242.1	CBV4/16JZ0613	China	Feline	2016	II	FBoV
MH155936.1	17CC0507	China	Feline	2017	II	FBoV
MK671247.1	17SY0602	China	Feline	2017	II	FBoV
MK671239.1	CBV4/17CC0505	China	Feline	2017	II	FBoV
MH155951.1	7CC0505-BoV2	China	Feline	2017	II	FBoV
MH155939.1	17CC0704-BoV2	China	Feline	2017	II	FBoV
ON595891.1	FPV-2	Australia	Feline	2017	II	FBoV
ON595892.1	FPV-3	Australia	Feline	2017	II	FBoV
MK671256.1	18QQHE0709	China	Feline	2018	II	FBoV
MK671253.1	CBV4/18HRB0502	China	Feline	2018	II	FBoV
MK671248.1	BV4/18BC0503	China	Feline	2018	II	FBoV
MK671258.1	CBV4/18SY0903	China	Feline	2018	II	FBoV
MK671250.1	CBV4/18CC0908	China	Feline	2018	II	FBoV
OR544345.1	NG/104	China	Feline	2021	II	FBoV
OR544346.1	DL/102	China	Feline	2022	II	FBoV
JQ692586.1	HK797U	China	Feline	2009	I	FBoV
JQ692587.1	HK875F	China	Feline	2010	I	FBoV
KP769860.1	MG132167B	Belgium	Feline	2013	I	FBoV
KM017745.1	FBD2	USA	Feline	2013	I	FBoV
MK671209.1	CBV3/16JL0904	China	Feline	2016	I	FBoV
MK671218.1	CBV3/17DD0504	China	Feline	2017	I	FBoV
MK671237.1	CBV3/18SY0102	China	Feline	2018	I	FBoV
MK671235.1	CBV3/18LY0504	China	Feline	2018	I	FBoV
MK671233.1	CBV3/18JL0603	China	Feline	2018	I	FBoV
MK671231.1	CBV3/18JL0502	China	Feline	2018	I	FBoV
MK671229.1	CBV3/18HRB1001	China	Feline	2018	I	FBoV
MK671201.1	CBV3/18CC0712	China	Feline	2018	I	FBoV
MK671236.1	CBV3/18QQHE0501	China	Feline	2018	I	FBoV
MK671202.1	CBV3/18HRB0506	China	Feline	2018	I	FBoV
MN127776.1	18R217C/THA	Thailand	Feline	2018	I	FBoV
MH155945.1	17JL0317	China	Feline	2017	I	FBoV
MH155947.1	17CC0302	China	Feline	2017	I	FBoV
MK671199.1	CBV3/17SY1201	China	Feline	2017	I	FBoV
MK671222.1	CBV3/17JL0317	China	Feline	2017	I	FBoV
MK671217.1	CBV3/17DD0501	China	Feline	2017	I	FBoV
MK671216.1	CBV3/17CC0505	China	Feline	2017	I	FBoV
MH155943.1	17HRB0910	China	Feline	2017	I	FBoV
MH155946.1	16SY0602	China	Feline	2016	I	FBoV
MH155931.1	16JL0804	China	Feline	2016	I	FBoV
MK671212.1	CBV3/16SP0902	China	Feline	2016	I	FBoV
MH155935.1	16SY0715	China	Feline	2016	I	FBoV
MK671213.1	CBV3/16SY0602	China	Feline	2016	I	FBoV
KX228695.1	HRB2015-LDF	China	Feline	2015	I	FBoV
KM017744.1	FBD1	USA	Feline	2013	III	FBoV
DQ474237.1	GT-3	China	Feline	2009	-	FPV
M38246.1	CU-4	USA	Feline	1996	-	FPV
AB000066.1	TU2	Japan	Feline	2009	-	FPV

FBoV=Feline bocavirus, FPV=Feline panleukopenia virus

### Recombination and statistical analysis

Potential recombination events among the FBoV strains were assessed using Recombination Detection Program (RDP v4.0) [28], applying multiple detection algorithms (BootScan, Chimaera, GENECONV, RDP, MaxChi, LARD, SiScan, 3Seq, and Phyl-Pro) with default parameters and a significance threshold of p < 0.01.

Statistical analyses were conducted using the Statistical Package for the Social Sciences software version 25.0 (IBM Corp., Armonk, NY, USA). Due to the small number of FBoV-positive cases, Fisher’s exact test was applied to assess associations between infection status and host variables. Statistical significance was determined at p < 0.05.

## RESULTS

### Detection of FBoV genome in feline fecal samples

Out of 166 fecal samples collected from domestic cats, FBoV genomic DNA was detected in 4 samples, corresponding to a positivity rate of 2.41%. The highest detection rates were observed in Hung Yen Province (4.17%) and Hanoi City (3.13%), whereas no FBoV-positive cases were identified in Bac Giang Province or Ha Nam Provinces (Table 3). With respect to clinical presentation, FBoV was detected in 6.67% of cats exhibiting gastrointestinal symptoms and 6.52% of clinically healthy cats. Notably, no positive cases were found in cats presenting with non-gastrointestinal clinical signs. Viral genomes were identified in both male and female cats, and across all age categories (<6 months, 6–12 months, and >12 months), although no statistically significant differences in detection rates were observed among these groups (Table 3).

**Table 3:** Detection of FBoVs in field samples using the PCR method.

Criteria	No. of tested samples	No. of gene-positive samples	Positive samples (%)	95% CI
Location				
Hanoi	64	2	3.13	0.38–10.80
Bac Giang	35	0	0	-
Hung Yen	48	2	4.17	0.51–14.25
Ha Nam	19	0	0	-
Clinical condition				
Gastrointestinal disorders	15	1	6.67	0.17–31.95
Other clinical signs	105	0	0	-
Heathy	46	3	6.52	0.13–17.90
Gender				
Male	93	1	1.08	0.03–5.85
Female	73	3	4.11	0.86–11.54
Age (months of age)				
<6	24	1	4.17	0.11–21.12
6–12	42	2	4.76	0.58–16.16
>12	100	1	1.00	0.02–5.45
Total	166	4	2.41	

FBoVs=Feline bocaviruses, PCR=Polymerase chain reaction, CI=Confidence interval

In addition, conventional PCR screening revealed that FBoV-positive samples were negative for other major feline enteric viruses, including FCoV, FeAstV, FKoV, and FPV. These additional findings are part of an ongoing investigation and will be reported separately.

### Genetic and phylogenetic characterization of FBoV strains

To elucidate the genetic characteristics of circulating FBoV strains, four FBoV-positive samples were selected for partial NS1 gene sequencing. These strains were designated as follows: Feline/Vietnam/FBoV1/V NUA-54/2023, Feline/Vietnam/FBoV1/VNUA-96/2023, Feline/Vietnam/FBoV1/VNUA-110/2023, and Feline/Vietnam/FBoV2/VNUA-101/2023 (Table 4). Pairwise nucleotide identity among the four strains ranged from 64.68% to 99.57%, with the highest similarity observed between strains VNUA-54/2023 and VNUA-96/2023, and the lowest between VNUA-110/2023 and VNUA-101/2023 (Table 5). When compared with reference strains in the GenBank database, sequence identity ranged from 98.58% to 99.71% (Table 6), indicating high genetic similarity to FBoV strains previously reported in China.

**Table 4:** Detailed information about the FBoV strains obtained in this study.

S. No.	Strain name	Location	Month of age	Sex	Clinical status	Genotype
1.	Feline/Vietnam/FBoV1/VNUA-54/2023	Hanoi	16	Female	Gastrointestinal disorders	I
2.	Feline/Vietnam/FBoV1/VNUA-110/2023	Hung Yen	8	Female	Healthy	I
3.	Feline/Vietnam/FBoV2/VNUA-101/2023	Hung Yen	10	Female	Healthy	II
4.	Feline/Vietnam/FBoV1/VNUA-96/2023	Hanoi	4.5	Male	Healthy	I

FBoV=Feline bocavirus, VNUA=Vietnam National University of Agriculture

**Table 5:** Comparison of the nucleotide identity of the partial NS1 gene (705 bp) among the sequences of four Vietnamese FBoV strains obtained in this study.

Strain name	Nucleotide identity (%)			

	1	2	3	4
Feline/Vietnam/FBoV1/VNUA-54/2023	100			
Feline/Vietnam/FBoV1/VNUA-110/2023	99	100		
Feline/Vietnam/FBoV2/VNUA-101/2023	64.82	64.68	100	
Feline/Vietnam/FBoV1/VNUA-96/2023	99.57	99.43	64.96	100

FBoV=Feline bocavirus, VNUA=Vietnam National University of Agriculture

**Table 6:** Comparisons of nucleotide identity of the partial NS1 gene (705 bp) of the sequences of four Vietnamese FBoV strains with downloaded sequences from the GenBank database.

Strain name	Strain	Country	Accession number	Year	Identity (%)
Feline/Vietnam/FBoV1/VNUA-54/2023	CBV3/16CC0803	China	MK671205.1	2016	99.71
Feline/Vietnam/FBoV1/VNUA-110/2023	CBV3/16CC0803	China	MK671205.1	2016	99.14
Feline/Vietnam/FBoV2/VNUA-101/2023	CBV4/18CC0908	China	MK671250.1	2018	98.58
Feline/Vietnam/FBoV1/VNUA-96/2023	CBV3/16CC0803	China	MK671205.1	2016	99.57

FBoV=Feline bocavirus, VNUA=Vietnam National University of Agriculture

**Figure 1:**
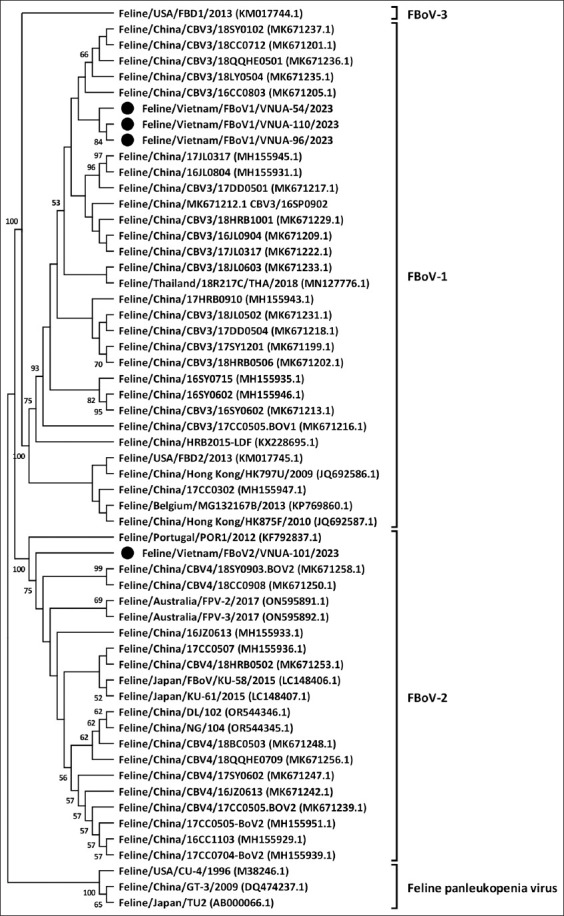
Maximum likelihood phylogenetic tree of partial NS1 gene (705 bp) sequences of Vietnamese feline bocavirus strains compared with those available in GenBank. The MEGA X software maximum likelihood method was used to construct a phylogenetic tree (1000 bootstrap replicates). Numbers at each branch indicate bootstrap values of .50% by the bootstrap interior branch test. The present Vietnamese strains are indicated by solid black circles.

Phylogenetic analysis based on a 705 bp fragment of the partial NS1 gene confirmed the presence of two distinct genotypes among the Vietnamese strains. Three isolates clustered within genotype I, while one isolate (VNUA-101/2023) grouped with genotype II (Figure 1). All Vietnamese strains shared close phylogenetic relationships with Chinese reference strains.

### Recombination analysis

Analysis of potential recombination events using the RDP v4.0 revealed no evidence of recombination among the Vietnamese FBoV strains. This suggests that the genetic diversity observed among these strains is primarily due to point mutations and evolutionary divergence, rather than recombination.

## DISCUSSION

This study aimed to investigate the molecular prevalence, genotype distribution, and genetic characteristics of FBoV strains circulating in domestic cats in Northern Vietnam.

### Circulation and genotypic diversity of FBoV in Vietnam

FBoV has been documented in various countries across Asia, including Japan, China, Taiwan, and Thailand [6, 10, 11], following its first identification in stray cats in Hong Kong in 2011 [9]. In contrast, Vietnam has thus far only reported HBoV infections in pediatric patients hospitalized with acute respiratory symptoms in southern provinces [3]. The present study represents the first molecular investigation of FBoV among domestic cats in Northern Vietnam and reports a detection rate of 2.41%. This prevalence is lower than that reported in previous studies from the United States (8%) [29], Portugal (5.5%) [12], Japan (9.9%) [11], and Northeast China (25.9%) [21]. Such discrepancies in prevalence may be attributed to regional ecological differences, temporal factors in sampling, and variation in detection methodologies.

Partial sequencing of the NS1 gene revealed that the four Vietnamese strains belonged to two distinct genotypes: Three to genotype I and one to genotype II. All strains showed high nucleotide identity to Chinese isolates, indicating probable transboundary transmission or shared evolutionary origins. The concurrent circulation of multiple genotypes, as also reported in China and Thailand [10, 21, 30], suggests that FBoV in Vietnam exhibits considerable genetic diversity. Although the existence of distinct serotypes has not been confirmed, the co-circulation of multiple genotypes necessitates further investigation to inform vaccine development, disease control strategies, and evolutionary studies.

### Clinical association and co-infection considerations

FBoV has been associated with various clinical manifestations, including hemorrhagic enteritis and neurological signs in cats [6, 30]. In this study, FBoV was detected in both symptomatic (diarrheic) and asymptomatic cats, consistent with earlier findings that highlight the variable clinical impact of different genotypes. Piewbang *et al*. [6] observed that FBoV-1 is more commonly detected in diarrheic cats, whereas FBoV-2 and FBoV-3 are frequently isolated from healthy animals. Similarly, our study detected a genotype I strain in a diarrheic cat and a genotype II strain in an asymptomatic cat, reinforcing the need to elucidate genotype-specific pathogenicity through controlled experimental studies.

Moreover, co-infection with other enteropathogens (e.g., FCoV, FPV, FKoV, FeAstV) is common in feline gastrointestinal diseases [31–36], yet none of the FBoV-positive cats in this study were co-infected with these viruses. This absence may indicate a sole viral etiology in these cases or may reflect limitations in detection sensitivity. Future studies should consider broader pathogen panels, including parasitic and bacterial agents, to better understand the etiological complexity of feline diarrheal diseases.

### Epidemiological factors and host susceptibility

Takano *et al*. [11] have found no significant association between FBoV infection and host factors such as age or sex, which aligns with our findings. The FBoV-positive cases in this study were distributed across all age groups and both sexes without statistical significance. This suggests that host demographic variables may not be primary determinants of FBoV susceptibility in domestic cats.

### Recombination and genomic insights

Recombination is a recognized mechanism driving genetic variability and the emergence of novel BoV strains, particularly within the NS1 region [6, 14]. While recombination events have been reported in Chinese FBoV isolates, no evidence of recombination was detected among the Vietnamese strains in this study using RDP4. Nevertheless, the limited number of sequences analyzed restricts the ability to draw firm conclusions, and future studies with expanded datasets are necessary to assess recombination dynamics more robustly.

## CONCLUSION

This study provides the first molecular evidence of FBoV circulation in domestic cats in Northern Vietnam, with an overall detection rate of 2.41% among 166 fecal samples collected from four provinces. Molecular characterization of partial NS1 gene sequences revealed the presence of two genotypes – FBoV-1 (three strains) and FBoV-2 (one strain) – highlighting the co-circulation of genetically distinct viral lineages. Phylogenetic analysis demonstrated close relatedness to previously reported Chinese strains, suggesting potential cross-border viral transmission or shared evolutionary origins. No recombination events were observed, and FBoV was detected in both diarrheic and asymptomatic cats, with no statistically significant association with age or sex.

The findings have several practical implications. First, the detection of multiple FBoV genotypes in clinically healthy and diseased cats underscores the necessity of including FBoV in differential diagnostic protocols for feline enteric diseases. Second, the absence of co-infections with other common feline enteric viruses (FCoV, FPV, FKoV, FeAstV) in positive cases suggests the potential for FBoV to act as a primary pathogen, although its pathogenicity requires further clarification. In addition, the observed genetic diversity should be considered in the context of vaccine development and molecular surveillance strategies.

A key strength of this study is its methodological rigor, employing validated molecular techniques and robust phylogenetic analysis to confirm the presence and diversity of FBoV strains. However, several limitations must be acknowledged. The study was geographically restricted to Northern Vietnam and had a modest sample size, potentially limiting the generalizability of the results. Moreover, genetic analysis was confined to partial NS1 sequences, precluding insights into the full genomic architecture and potential recombination patterns.

Future research should expand sampling across broader geographic regions and timeframes, incorporate whole-genome sequencing, and investigate other relevant viral genes such as VP1, VP2, and NP1. Experimental infection studies and seroepidemiological surveys are also warranted to elucidate genotype-specific virulence and host immune responses.

In conclusion, this study establishes a foundational understanding of FBoV epidemiology in Vietnam and underscores the need for continued surveillance and comprehensive molecular characterization to inform disease management, improve diagnostic capabilities, and anticipate viral evolution in domestic feline populations.

## AUTHORS’ CONTRIBUTIONS

HVD, DATB, AR, CB, and JR: Designed the study. HVD, GTHT, YHTN, and TCN: Sample collection. HVD, TCN, AR, CB, JR: Performed the study. HVD, AR, CB, JR: Data analyses. HVD, AR, CB, and JR: Drafted and revised the manuscript. All authors approved the finalized manuscript.

## Data Availability

All the generated data are included in the manuscript.
